# Feasibility and safety of one-stage sacral laminoplasty with autologous sacral laminar reimplantation fixed by absorbable fixation clamps in direct microsurgical treatment of symptomatic sacral extradural spinal meningeal cysts

**DOI:** 10.3389/fsurg.2023.1253432

**Published:** 2023-11-21

**Authors:** Xiaoliang Yin, Jia Zhang, Qianquan Ma, Suhua Chen, Chao Wu, Chenlong Yang, Yu Si, Haihui Jiang, Wei Guo, Ying Liu, Huishu Yuan, Jun Yang, Jianjun Sun

**Affiliations:** ^1^ Department of Neurosurgery, Peking University Third Hospital, Peking University, Beijing, China; ^2^ Department of Radiology, Peking University Third Hospital, Peking University, Beijing, China; ^3^ Department of Neurosurgery, Beijing Friendship Hospital, Capital Medical University, Beijing, China

**Keywords:** sacral extradural spinal meningeal cysts, microsurgical treatment, sacral laminoplasty, sacral lamina, absorbable fixation clamps

## Abstract

**Introduction:**

Sacral laminoplasty with titanium mesh and titanium screws can reduce symptomatic sacral extradural spinal meningeal cysts (SESMCs) recurrence and operation complications. However, due to a defect or thinning of the sacrum, the screws cannot be securely anchored and there are also problems with permanent metal implantation for titanium mesh and screws. We propose that sacral laminoplasty with absorbable clamps can provide rigid fixation even for a thinned or defected sacrum without leaving permanent metal implants.

**Methods:**

In the direct microsurgical treatment of symptomatic SESMCs, we performed one-stage sacral laminoplasty with autologous sacral lamina reimplantation fixed by absorbable fixation clamps. Retrospectively, we analyzed intraoperative handling, planarity of the sacral lamina, and stability of the fixation based on clinical and radiological data.

**Results:**

Between November 2021 to October 2022, we performed sacral laminoplasty with the absorbable craniofix system in 28 consecutive patients with SESMCs. The size of the sacral lamina flaps ranged from 756 to 1,052 mm^2^ (average 906.21 ± 84.04 mm^2^). We applied a minimum of two (in four cases) and up to four (in four cases) Craniofix clamps in the operation, with three (in 20 cases) being the most common (82.14%, 20/28) and convenient to handle. Excellent sacral canal reconstruction could be confirmed intraoperatively by the surgeons and postoperatively by CT scans. No intraoperative complications occurred.

**Conclusions:**

One-stage sacral laminoplasty with absorbable fixation clamps is technically feasible, and applying 3 of these can achieve a stable fixation effect and are easy to operate. Restoring the normal structure of the sacral canal could reduce complications and improve surgical efficacy.

## 1. Introduction

Sacral extradural spinal meningeal cysts (SESMCs) are extradural meningeal cysts located in the sacral canal (1, 2). The most accepted theory is the ball-valve mechanism, which is attributed to congenital dysplasia or acquired trauma, inflammation and other factors, arachnoidal defect, and a one-way valve that communicates with the subarachnoid space formed (3, 4). Driven by pulsatile and hydrodynamic forces, cerebrospinal fluid flows in one direction, resulting in the formation and continuous expansion of SESMCs (3, 5). The enlargement of the SESMCs can exert a significant mass effect due to their elevated internal pressure, resulting in compression of the surrounding neural tissue and gradual damage to the adjacent bone (6–8).

In the past, the sacral lamina was not considered a crucial structure, and sacral laminoplasty was rarely been performed. However, recent evidence has shown that laminoplasty may reduce postoperative CSF leakage rates and mitigate epidural fibrosis (9–11). Chen et al. reported that sacral laminoplasty using titanium mesh and titanium screws effectively lowered the recurrence of SESMCs and minimized complications related to cerebrospinal fluid leakage (9). Nevertheless, in cases involving severe erosion of the dorsal sacral wall, resulting in a substantial defect or thinning of the sacral canal, securely anchoring screws can be problematic. Additionally, metal implants may introduce artifacts in MRI images.

**Figure 1:**
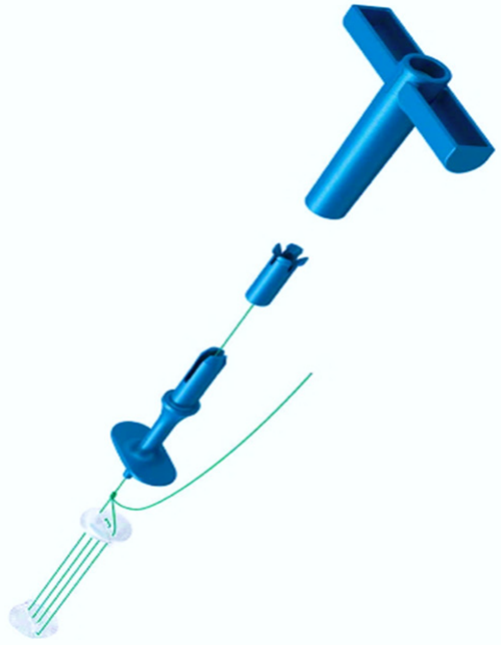
Schematic illustration of the absorbable Craniofix structure.

Craniofix® Absorbable (FF017, Aesculap, AG, Tuttlingen, Germany) (Figure 1) is a type of fixation clamp constructed from absorbable polyester, widely utilized for the fixation of the cranial flap after craniotomy (12, 13). We performed sacral laminoplasty with absorbable fixation clamps in direct microsurgical treatment of SESMCs and obtained satisfactory fixation effect and good clinical efficacy, even in cases involving bone defects. To the best of our knowledge, this is the first reported clinical application of the absorbable Craniofix technique for sacral laminoplasty.

## 2. Methods

### 2.1. Study design

**Figure 2:**
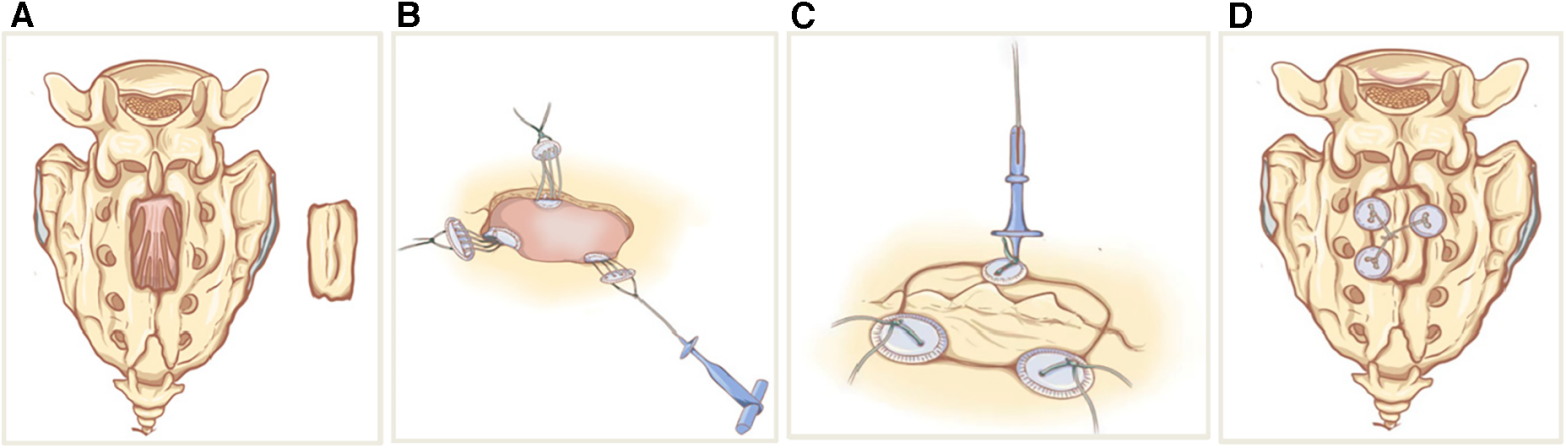
Schematic sketches showing the principle of sacral laminoplasty with absorbable fixation clamps. (**A**) The bilateral laminae sacrum was incised by ultrasonic bone scalpel(UBS) to form an integral spinous process-lamina complex bone flap. (**B**) The bottom clamps of the absorbable craniofix position inferior to the edge of the sacral bone window and place the upper clamp outside. (**C**) The spinous process-lamina complex bone flap is inserted and the upper clamps are drawn down by pulling the prepared sutures in the manner of a chain block. (**D**) The prepared sutures on the opposite sides are crossed and fixed with knots so the sacral lamina is rigidly refixed with carniofixes.

Between November 2021 to October 2022, we conducted 28 direct microsurgical treatments for SESMCs. We carried out a clinical assessment to determine the feasibility and safety of sacral laminoplasty using absorbable Craniofix (Figure 2). Approval for the study was obtained from the Research Ethics Board of Peking University Third Hospital. All subjects provided written informed consent for surgical procedures. All surgeons possessed extensive experience in performing absorbable Craniofix procedures for cranioplasty before transitioning to sacral laminoplasty.

**Figure 3:**
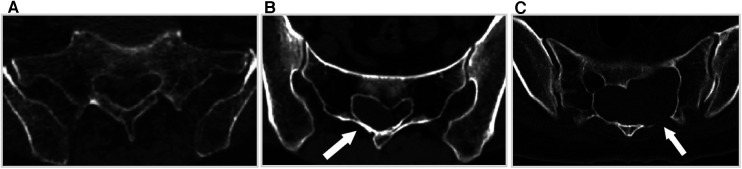
The integrity scale of the sacral lamina. (**A**) The sacral lamina is intact. (**B**) The sacral lamina is eroded and thinning (white arrow). (**C**) The sacral lamina is partial full-thickness defect (white arrow).

Patients were included in the study if they met the following criteria: (1) MRI results confirming the presence of SESMCs, (2) neurological symptoms attributed to SESMCs, (3)the presence of symptoms lasting for a period of at least 6 months, and (4)severe pain refractory to medical treatment. Preoperative CT scans of the sacral canal were conducted to evaluate the integrity of the sacral lamina on scale 1 (intact) (Figure 3A) to scale 2(eroded thinning) (Figure 3B) and scale 3 (full-thickness defect) (Figure 3C). Patients were excluded from the study if their symptoms could not be distinguished from lumbar spinal stenosis or lumbar intervertebral disc herniation. All patients included in this study provided informed consent and signed an acceptance letter.

### 2.2. Microsurgical management

**Figure 4:**
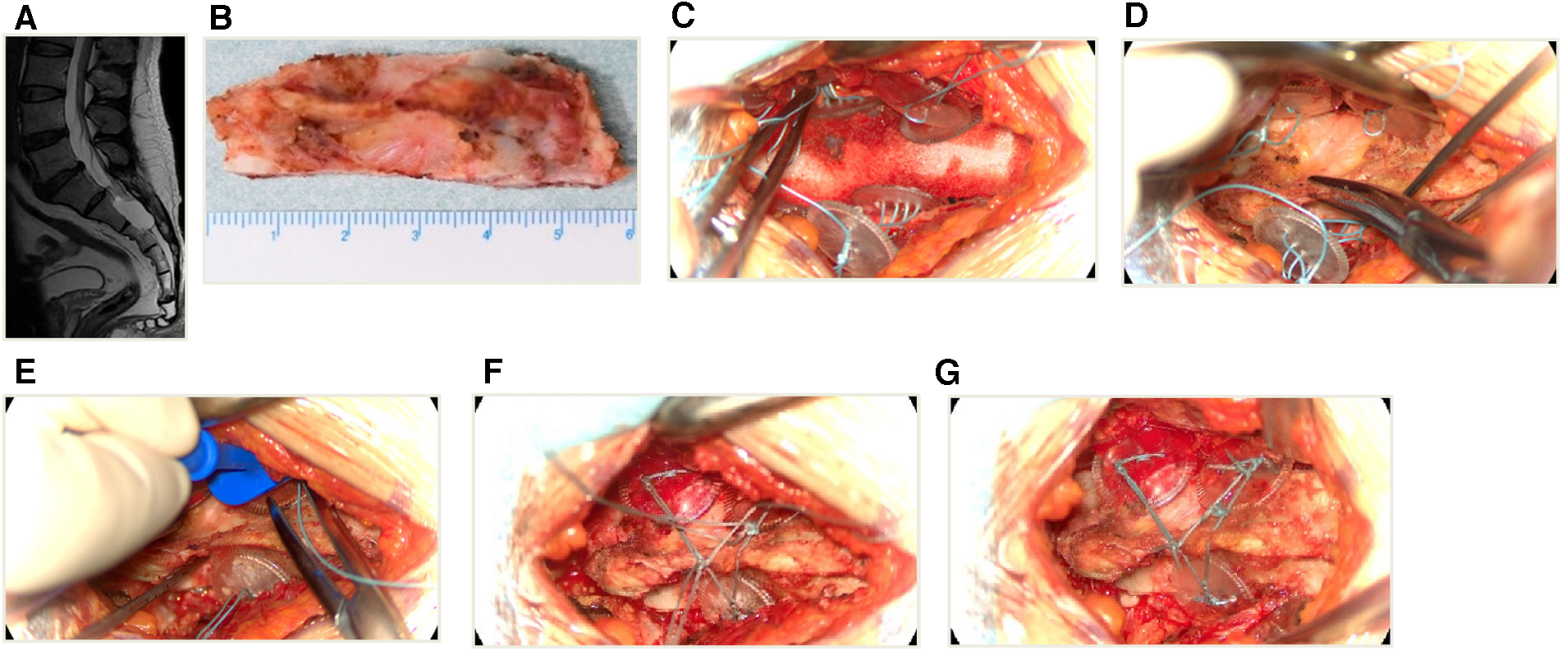
An illustrative case of sacral laminoplasty with absorbable fixation clamps. (**A**) The preoperative MRI T2-weighted image of a SESMC case. (**B**) The bilateral laminae were opened with UBS and the entire spinous process-lamina complex was removed. (**C**) Placement of three absorbable Craniofixes with one on the left and two on the right side of the sacral lamina bone window. (**D**) The sacral lamina bone flap was inserted. (**E**) The upper clamp was drawn down by pulling the prepared suture in the manner of chain block plates. (**F**) The prepared sutures on the opposite sides are crossed and fixed with knots. (**G**) The excess sutures were cut off and the sacral lamina was fixed with excellent planarity and stability.

A comprehensive description of the microsurgical procedures has been previously documented in our published articles (14, 15). A brief summary of the procedure is as follows: Under general anesthesia, patients were positioned in the prone waist-bridge posture with the head facing downward. Real-time monitoring of somatosensory evoked potential (SEP) and motor evoked potential (MEP) was conducted. According to the location of the cyst on the preoperative MRI scan (Figure 4A), a corresponding skin incision was made in the posteromedial line and the paraspinal muscles were dissected along the midline to expose the sacral spinous process and bilateral fused lamina. The bilateral laminae were meticulously opened using an ultrasonic bone scalpel (UBS) (BoneScalpel, Misonix, Farmingdale, New York), removing the entire spinous process-lamina complex(Figure 4B) with care to preserve the integrity of the cyst wall. Preoperative MRI findings were confirmed under a microscope. Cysts with nerve root passage underwent nerve root sheath reconstruction, and cysts without nerve roots received neck transfixion.

### 2.3. Sacral laminoplasty and intraoperative assessment

Following the completion of the aforementioned procedure, the reconstruction was executed as follows. We usually use a minimum of two and a maximum of four absorbable Craniofixes on the left and right sides of the sacral lamina. First, we sequentially insert half of the bottom clamp inferior to the edge of the sacral bone window and place the upper clamp outside(Figure 4C), thereafter the sacral lamina bone flap is inserted (Figure 4D). Once the sacral lamina bone flap securely fits the bone window, the upper clamp is positioned against the lower clamp and tightened by pulling the prepared suture in the manner of a chain block until the sacral lamina bone flap is clamped firmly (Figure 4E). Since the traction torque is predefined by the disruption of the handle at a predetermined breaking point, the handle is broken by increasing the pulling force. Following this, three knots are tied using the prepared sutures to lock the absorbable Craniofix (Figure 4F). Finally, the prepared sutures on the opposite side are intertwined, secured with knots, and subsequently trimmed (Figure 4G), before the final step of wound closure.

The intraoperative records encompassed several key aspects, including the count and placement of absorbable Craniofixes, an assessment of the planarity and stability of the bone flap, and the time required for sacral laminoplasty. The intraoperative planarity scale ranged from scale 1 to scale 3 (as detailed in Table 1). Both the subjective rating of the stability of the bone flap on a scale from 1 (properly fixed) to 5 (loose) and the subjective rating of the handling of the clamps on a scale from 1 (very good) to 5(complicated) was evaluated by the surgeons (Table 2).

**Table 1:** Planarity scale of sacral laminoplasty.

Scale	Criteria	Diagram
1	The edges of the bone window and the bone flap are well aligned without displacement.	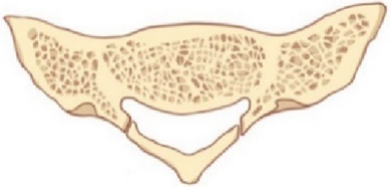
2	The edges of the bone window and the bone flap are slightly uneven, but the subsidence depth of the bone flap is less than the thickness of the sacral lamina.	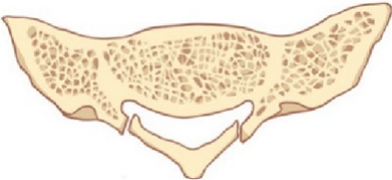
3	The edges of the bone window and the bone flap are detached, and the subsidence depth of the bone flap is greater than the thickness of the sacral lamina.	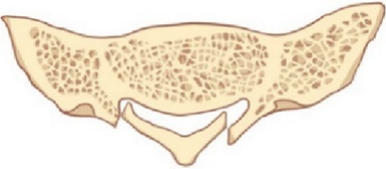

**Table 2:** Subjective ratings evaluated by the surgeons.

Aspect	Rating Scale	Description
Bone Flap Stability	1	Properly fixed
	2	Mostly stable
	3	Moderately stable
	4	Somewhat loose
	5	Loose
Clamps Handling	1	Very good
	2	Good
	3	Average
	4	Challenging
	5	Complicated

### 2.4. Postoperative management and assessment

**Figure 5:**
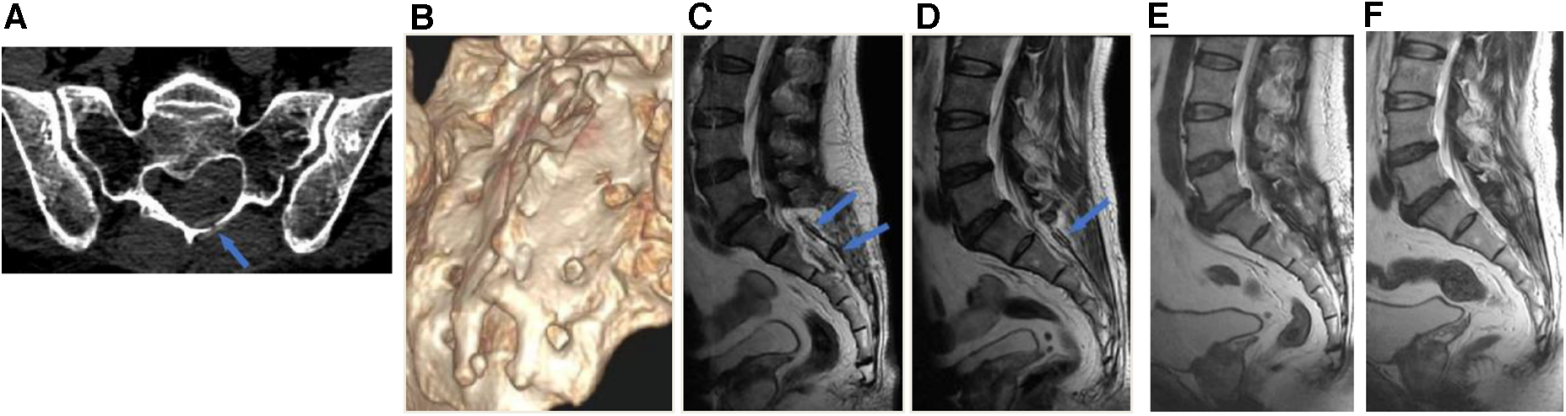
Postoperative radiological assessments. (**A**) The absorbable clamp (arrow) can be seen at the left side of the laminotomy on the postoperative axial CT image. (**B**) Postoperative 3D-CT showed sacral laminoplasty with excellent planarity and stability. (**C**) Postoperative 2-week MRI T2-weighted image showed 2 absorbable carniofixes at the right side of sacral laminectomy (blue arrows). (**D**) Postoperative 2-week MRI T2-weighted image showed 1 absorbable carniofix at the left side of sacral laminectomy (blue arrow). (**E and F**) Postoperative 6-month MRI revealed the bone structure of the sacral canal posterior wall remained stable, in comparison to the MRI at 2 weeks post-surgery, there was a significant improvement in the reduction of soft tissue swelling in the para-vertebral region.

Postoperatively, all patients remained in a prone position for several days. Wound healing was assessed and categorized as either progressing well, experiencing delayed healing, or requiring debridement and suturing. Sacral canal CT scans were obtained on average 8 (6–9) days after the operation, from which we evaluated the planarity of the bone flap and calculated the size (Figures 5A,B). Additionally, MRI scans were performed 2 weeks post-surgery (Figures 5C,D), and follow-up MRI scans were performed 6 months after surgery (Figures 5E,F). Postoperative radiological evaluation of the sacral canal was performed by a neuroradiologist who remained blinded to the patient's intraoperative diagnosis. Results were classified into three categories: complete cyst resolution, residual cyst, or disappearance of cysts with effusion into the canal cavity.

## 3. Results

From November 2021 to October 2022, 28 patients ranging from 26 to 75 years of age (mean 42.18 ± 15.49 years) with SESMCs underwent direct microsurgery and the absorbable Craniofix system was used for sacral canal closure. Of all the patients, 67.86% (19/28) were female, and 32.14% (9/28) were male. The lesions were located at S1–S5, where S2 was the most common site. Notably 92.86% (26/28) of the cysts involved more than one vertebral segment, as detailed in Table 3. Preoperative CT scans showed sacrum involvement in a total of 18 (64.29%, 18/28)patients, including 10 (35.71%, 10/28) patients with eroded thinning (scale 2) and 8 (28.57%, 8/28) patients with full-thickness defect (scale 3) respectively.

**Table 3:** Consecutive cases of SESMCs treated with sacral laminoplasty with autologous sacral laminar reimplantation fixed by absorbable fixation clamp.

Case	Age/Gender	Lesion Spinal-level	Sacrum integrity preop. CT [1–3]	Craniofix clamps [number]	Handling [1–5]	Stability [1–5]	Planarity intraop. [1–5]	Planarity postop. CT [1–5]	Bone flap area postop. CT (mm^2^)
1	55/F	S1-S2	1	2	1	2	1	1	795
2	35/M	S2-S3	3	3	2	1	1	1	1,052
3	61/F	S2-S4	2	3	1	4	2	2	851
4	16/F	S1-S3	1	4	3	1	1	1	789
5	45/F	S2	1	2	1	2	2	1	765
6	20/F	S2-S3	1	3	2	1	1	1	862
7	33/M	S1-S4	3	3	1	1	1	1	843
8	45/F	S1-S2	3	3	1	1	1	1	906
9	61/F	S2-S3	1	3	1	1	1	1	796
10	35/M	S1-S2	2	4	2	1	2	1	842
11	42/F	S2-S3	2	3	1	2	1	1	892
12	28/M	S1-S3	2	3	1	1	1	1	762
13	31/F	S2-S4	3	3	1	2	1	1	962
14	30/M	S2-S4	3	3	1	2	1	1	896
15	58/F	S1-S2	3	3	2	2	2	2	952
16	47/F	S2-S5	1	2	1	2	2	1	756
17	31/M	S2-S4	2	3	1	1	1	1	896
18	65/F	S3	1	2	1	1	1	1	964
19	64/F	S2-S3	2	3	2	2	2	2	936
20	36/M	S1-S3	2	4	2	1	1	1	968
21	31/F	S1-S5	2	3	1	2	1	1	986
22	29/F	S2-S3	1	3	2	1	1	1	889
23	75/F	S1-S2	2	3	1	1	1	1	979
24	23/M	S2-S3	3	3	1	3	1	2	963
25	50/F	S1-S3	3	4	3	2	1	1	1,025
26	66/M	S2-S3	1	3	1	1	1	1	882
27	39/F	S2-S4	1	3	1	2	1	1	959
28	30/F	S1-S3	2	3	1	1	1	1	906

preop, preoperative; intraop, intraoperative; postop, postoperative; CT, computed tomography.

We did not observe intraoperative complications, nor did we need to change the method in any patient since the absorbable Craniofix clamp can be successfully applied to sacral canaloplasty with good fixation even for partial full-thickness sacral defects. We applied a minimum of two (in four cases) and up to four (in four cases) Craniofix clamps in the operation, with three (in 20 cases) being the most common (82.14%, 20/28). The procedure time for sacral laminoplasty ranged from 12 min to 16 min (average 14.16 ± 1.26 min). The size of the sacral lamina flaps ranged from 756 to 1,052 mm^2^ (average 906.21 ± 84.04 mm^2^±) (Table 1). The average value of handling the clamps, subjectively evaluated by the surgeon, was 1.42 ± 0.73 (Table 2). The average stability scale of the bone flap, subjectively evaluated by the surgeon, was 1.57 ± 0.73 (Table 3). The average planarity scale was 1.21 ± 0.32 intraoperatively evaluated by the surgeon and 1.14 ± 0.35 postoperatively evaluated by CT images. The postoperative period was uneventful and the wounds healed well and postoperative MRI scans on 2 weeks showed total resection of cysts without pseudocysts and effusions in all cases (as illustrated in Figures 5C,D). At the 6-month postoperative follow-up, MRI scans revealed secure and stable bone structures of the sacral canal posterior wall, with no signs of abnormal reactions or inflammation around the fixation material. Furthermore, in comparison to the MRI scans at 2 weeks, there was a noticeable improvement in the reduction of soft tissue swelling in the para-vertebral region. The sacral canal cyst did not reoccur, and there were no complications such as subcutaneous fluid accumulation or pseudocysts (as illustrated in Figures 5E,F).

## 4. Discussion

### 4.1. Benefits of sacral laminoplasty

Direct microsurgical treatment of SESMCs has demonstrated efficacy in previous studies by us and other authors (16–19)and is therefore recommended. Techniques for nerve root and terminal cistern reconstruction, including overlapping nerve roots with the cyst wall, redundant cyst wall excision, and strengthening of the reconstructed nerve sheath with an artificial dura mater, have been discussed in depth in our previous studies (14, 15). This study mainly focuses on the reconstruction of the sacral canal bone structure.

In the past, the sacral lamina was not considered an essential structure, and sacral laminoplasty was rarely performed (9). However, when the sacral canal is filled with muscle or fat without dorsal wall reconstruction, it can lead to the formation of numerous epidural scars within the sacral lamina defect area. This may result in nerve adhesion and secondary spinal stenosis, and even in the recurrence of nerve compression symptoms or aggravation of the original symptoms (20). Recent evidence suggests that laminoplasty can reduce postoperative CSF leakage rates and diminish epidural fibrosis. Smith et al. (11). reported sacral laminoplasty by reimplantation of the removed lamina fixed by titanium mini-plates to treat 18 symptomatic sacral perineural cyst patients. No CSF leakage and no radiographic recurrence at 12 months follow-up was found. Chen et al. (9). performed sacral laminoplasty with a titanium mesh to cover the sacral laminal defect. The titanium mesh was shaped according to the size and contour of the sacral laminal defect and fixed to the sacrum with screws (length 3–5 mm). During follow-ups, ranging from 13 to 37 months, all six patients experienced no recurrence of dural ectasia or pseudomeningocele and were free from local symptoms. In our study of the 28 consecutive cases, the additional reconstruction of the sacral canal posterior wall took approximately 12–16 min, which did not significantly extend the overall surgical duration. Nevertheless, this supplementary step improved the surgical results significantly. The postoperative period was uneventful, and an MRI scan showed the complete resection of SESMCs without cerebrospinal fluid leakage, pseudocyst formation, or effusion in all cases.

### 4.2. Advantages of Craniofix for different bone defects

Based on our previous institutional experience (21), we found that sacral laminoplasty using titanium plates or titanium mesh achieved satisfactory outcomes for small cysts with intact sacral canal bones. However, for large cysts, the sacral canal bone is often eroded, thinned, or defective, making it challenging to achieve reliable fixation. The mechanism of Craniofix relies on the firm clamping of the sacral lamina between the two clamps and it is not affected by the thickness of the sacral lamina. Therefore, sacral laminoplasty with absorbable Craniofix fixation is feasible no matter whether there are intact, eroded, or partial full-thickness defect bones. Our results confirmed that all patients can obtain good spinal canal reconstruction and no case needed a change to the surgical method. Especially for those with bone defects, similar to the fixation in the burr hole area of the skull, the application of the cranial clip can also partially cover the sacral bone defect area. This effectively reconstructed the posterior wall of the sacral canal, removed the sacral canal cyst and subcutaneous cyst, and closed the fistula between them, significantly reducing the recurrence rate.

### 4.3. Optimal implant number and stability assessment

Different from cranial bone flap fixation, the position of the sacral lamina is deep and the operating space is relatively narrow. Based on our experiences with the absorbable Craniofixes, they were convenient to handle when we used up to two or three implants. The use of four Craniofixes tends to entwine with each other, making the operation inconvenient. Lemcke et al. (12). reported that three Craniofixes ensured sufficient stability for free bone flaps up to approximately 3,000 mm^2^. The size of the sacral bone flaps we fixed ranged from 756 to 1,025 mm^2^. Therefore, in most cases (20/28) we applied three implants, which is not only convenient for operation but also achieves a firm fixing effect. The stability and planarity were adequate in all of our patients evaluated intraoperatively by the surgeons and postoperatively by the radiologists.

### 4.4. Limitations of the study and outlook

Although our study successfully investigates the use of absorbable Craniofixes in sacral lamina fixation, this study has some limitations. First, this was a retrospective single-center study, which may limit the generalizability of the findings. Additionally, the sample size was relatively small, which could affect the statistical power of the result. Finally, as the technical feasibility and safety are from our consecutive cases, the lack of a control group is also a limitation of our study. Therefore, prospective controlled studies are needed to evaluate the exact effect of sacral laminoplasty with absorbable Craniofixes in direct microsurgical treatment of symptomatic sacral extradural spinal meningeal cysts.

## 5. Conclusions

One-stage sacral laminoplasty with absorbable fixation clamps is technically feasible and safe. The use of three such clamps can achieve a stable fixation effect and is easy to perform. By restoring the normal structure of the sacral canal, this procedure can reduce complications and improve surgical efficacy. However, due to the small sample size of consecutive cases, a final conclusion needs the accumulation of cases and be confirmed by prospective controlled studies.

## Data Availability

The original contributions presented in the study are included in the article/supplementary material, further inquiries can be directed to the corresponding author.
